# Supplementary material for: Small and Random Peptides: An Unexplored Reservoir of Potentially Functional Primitive Organocatalysts. The Case of Seryl-Histidine
Source: Life (Basel). 2017 Apr 9;7(2):19. doi: 10.3390/life7020019 (PMC5492141; doi:10.3390/life7020019)
Supplement: Supplementary File 1 [file life-179962-coverletter.pdf › life-179962-review report/life-179962-coverletter.pdf]

UNIVERSITÀ DEL SALENTO

Di.S.Te.B.A.
DIPARTIMENTO DI SCIENZE E TECNOLOGIE BIOLOGICHE ED AMBIENTALI

Pasquale Stano, Laboratory of Organic Chemistry
DiSTeBA, University of Salento; Campus Ecotekne, I-73100 Lecce, Italy
Phone (+39) 0832 298709, Fax (+39) 0832 298732, e-mail: pasquale.stano@unisalento.it

To the Editorial Office

Life – MDPI

Manuscript life-179962 (revised version)

Dear Anna,

we are grateful to the referees for their suggestions, as we had the chance of improving the manuscript and better discussing important aspects which were only shortly mentioned in the first submission.

Please find enclosed the new version of our manuscript (pdf). The changes, both scientific and linguistic have been evidenced in the accompanying file, attached to this letter (although it includes some LaTeX commands, it is anyway very readable).

Below, instead, you find our point-by-point replies to the reviewers' comments.

With kind regards,

Pasquale Stano, on behalf of all authors.

The new version has been corrected for the English by a native speaker as suggested by the **third reviewer**. Also all the remarks made by the first and the second reviewers have been addressed.

Reviewer 1:

1) There are a couple of mistakes regarding the use of “symbol” characters: page 2, line 60; and page 4, line 119.

The above-mentioned symbols have been corrected.

2.1) His is not in the list of “prebiotic amino acids” that the authors give, but it is crucial in the catalysts they study. Can the authors propose, of course based on the literature, any possible pathway to His, or some possible source of it?

This is an important remark, done also by Reviewer 2. Therefore we have added a new paragraph, with references to original works, discussing the prebiotic synthesis and availability of histidine. This paragraph can be found in the chapter “3. Origin of peptides”. In particular, we explicitly mentioned that His is not straightforwardly obtained by Strecker-like synthesis, but also cite relevant classical works by J. Oró that specifically deal with the subject.

2.2) In their revision of prebiotic syntheses toward oligopeptides, there are important recent papers missing: Murillo-Sanchez et al., 2016, *Chem. Sci.* **7**, 3406; Jaker et al., 2015, *Angew. Chem. Int. Ed.* **54**, 14564. These two papers are actually important because they treat oligopeptides in interaction with lipid membranes and oligonucleotides, respectively.

Those two papers have been added and briefly discussed in the chapter “3. Origin of peptides”.

2.3) In page 5, paragraph 3, the authors parallel experimental research lines toward oligopeptide libraries with Kaufmann´s ideas on autocatalytic chemical networks. This is of course reasonable, but a great limitation of their model is that in most of the peptide prebiotic syntheses described up to know, only dimers and trimers are obtained. This limits significantly their possible functions, and so such current limitation should be expressed more clearly somewhere in the review.

This limitation in respect to discussed Kauffman-like process has been clarified when it is discussed in the chapter “3. Origin of peptides”, in particular evidencing how longer peptides would be required for the establishment of a Kauffman-like scenario.

2.4) Something that is completely missing in the review relates to the work developed on peptide-based replication networks, by Ghadiri and Ashkenasy, among others, and which is fundamental if

one wants to parallel the proposed research line with Kaufmann´s models. Some of the Ashkenasy´s systems are actually catalytic: see e.g., Chem. Eur. J. 2016, v. 22, 6687.
We thank the referee for pointing out to this beautiful chemistry. As we tried to stay focused on the formation of peptide bonds, we did not include this work in the original version. However we agree with the referee that a short mention should be inserted for the sake of completeness. We have therefore added to the chapter “3. Origin of peptides” a paragraph referencing and briefly discussing the work on helical peptide replicators made by Ghadiri, Ashkenasy and others.

3) Another current limitation in the route to the peptide world suggested by the authors, which should be express more clearly, is that most of dimers, trimers and tetramers up to know are obtained from protected amino acid monomers. This raises doubts about the efficiency of the chemical couplings connecting oligopeptides that should lead to the proposed “fragment condensation scenario”. The authors should elaborate more on this, otherwise this scenario is for the moment not really credible.
The clarification required by the reviewer has been added to the discussion of the fragment condensation scenario in the chapter “5. A peptide fragment condensation scenario”. In particular, it has been evidenced that a realistic scenario should involve non-protected peptides (mentioned, however, in Figure 5, last line). Thanks to this specification, we give to the readers a correct vision of the fragment condensation scenario and the limitations of current approaches.

4) In pages 8-9, the authors are not very clear about the systems they refer to in the last paragraph of page 8 (peptides, right?) and in the first paragraph of page 9 (peptide nucleic acids, right?). This misunderstanding occurs in other parts of the review (e.g., last paragraph of page 12), and should be better clarified, as they are very different systems, with the only similarity that they are constituted by amide bonds.
Statements claryfying which compound is discussed has been added to the paragraphs in question in chapters “4.2 Peptide bond formation” and “4.4 A tentative discussion on the catalytic mechanism of SerHis”. Moreover we have modified Figure 5 to specify that the possibility of using peptide or PNAs building block only refers to the last line of the table. We thank the referee for noting this source of possible misunderstanding.

5) Finally, the catalytic triad Ser-His-Asp is mentioned. A pertinent question in this respect is whether it could have similar catalytic roles in prebiotic chemistry, considering its ubiquity in hydrolytic enzymes. Some further elaboration in this respect would be nice.

As far as the tested reactions allow to say Ser-His-Asp have all the functions present in Ser-His. In fact Ser-His is believed to work withouth the Asp moiety because the C-end carboxyl of His is available instead of Asp chain carboxyl. Therefore all that was said about Ser-His should be also true for Ser-His-Asp. We have added this discussion to the chapter “4.4 A tentative discussion on the catalytic mechanism of Ser-His”.

Reviewer 2:

His is considered a “phase 2” amino acid – i.e. it is an amino acid that does not appear to be synthesized easily by prebiotic chemical processes, and therefore requires the establishment of biosynthetic pathways to be created. Thus, its relevance for transitional chemistry in the origin of life is questionable.

As specified in the reply to the first reviewer, who raised a similar question, a new paragraph discussing the prebiotic synthesis and availability of histidine has been added to the chapter “3. Origin of peptides”.

The authors describe a molecular dynamics study of the Ser-His dipeptide (as well as the apparently non-functional His-Ser dipeptide) in order to identify a conformation that provides a plausible explanation for nucleophile activation of the Ser. Such studies fail to identify a structural basis for catalysis. This is somewhat problematic since the model system (a dipeptide) is comparatively simple and should permit adequate MD sampling to identify low energy conformations. Thus, the failure of the MD study calls the catalytic activity of Ser-His into question.

A recent report on the evaluation of catalytic properties for the Ser-His dipeptide by Gellman and coworkers refutes the claim of catalytic activity for the Ser-His dipeptide.

However, each of these issues is mentioned in the review by Stano; thus, while there is controversy regarding the subject, the authors review stands as a scholarly work that notes such objections. The review includes many appropriate references. Thus, the review is a scholarly work on a controversial subject.

We thank the referee for considering our work as impartial and well-balanced, reporting also results that are not in agreement with the main thesis. We believe that this is a good stimulus for carryng out more studies on this subject – actually an important theme in the origin of life investigation.

ABSTRACT

\abstract{Catalysis is an essential feature of living systems biochemistry, and probably it ~~has~~ played a key role in primordial times, helping to produce more complex molecules from simple ones. However, in such an ancient context, enzymes -- the biocatalysts \textit{par excellence} -- were not available. Rather, small molecule catalysis (organocatalysis) ~~could~~ may have occured. The best candidate ~~to~~for the role of primitive organocatalysts are amino acids and short random peptides, which are ~~supposed~~believed to have ~~to be~~been available ~~in the~~ on early Earth. In this review, we discuss the occurrence of primordial organocatalysts in the form of peptides, in particular commenting on intriguing reports about seryl-histidine dipeptide, which has been investigated recently. Starting from this specific case we also mention a peptide fragment condensation scenario, as well as other potential roles of peptides in primordial times. The review actually ~~points~~ aims to stimulate further investigation on an essentially unexplored field of research, namely ~~that~~ one that specifically looks at the catalytic activity of small random peptides with respect to reaction~~ of~~s relevan~~ce for~~t to prebiotic chemistry and early chemical evolution.}

\section{Introduction}

\label{sect:introduction}

In addition to inorganic factors, such as mineral surfaces \cite{lambert_adsorption_2008,sawai_prebiotic_1975}, light \cite{chittenden_prebiotic_1981}, reducing environment of hydrothermal vents \cite{cleaves_evaluation_2009}, and spark discharges \cite{bada_new_2013}, the role of organic molecules as early catalysts has been widely recognized in origin of life scenarios. Catalysis is omnipresent in modern life, and it might be argued that catalysis was one of the key mechanisms contributing to ~~life origin~~the origins of life. However, it is not easy to determine which were the primitive catalysts and what was their role, as well as in which phase of prebiotic evolution they emerged and how they contributed to the path, from the primordial chemical world to the birth of the first living cells.

In this respect, ~~it has been often discussed~~ the roles of small organic molecules which arose in abiotic syntheses \cite{sutherland_prebiotic_1997}, RNA \cite{derose_two_2002} or other simple nucleic acids \cite{schwartz_speculation_1997}, and small peptides \cite{szathmary_origin_1999,plankensteiner_prebiotic_2005,li_aminoacylating_2013}, whose chemical formation is viable under various routes \cite{parker_plausible_2014} have been discussed. The catalytic role of RNA in the modern ribosome has been adopted to validate the theory of a pure `RNA World' \cite{gilbert_origin_1986}, but the well-known chemical instability of RNA suggests more complex scenarios where the co-evolution of RNA and small peptides is depicted \cite{kunin_system_2000,li_aminoacylating_2013}. For example, recent theoretical investigations based on the activity and function of aminoacyl-tRNA suggest that an RNA-only metabolism ~~hardly~~ may never have existed \cite{van_der_gulik_how_2015}.

Since the 1953 seminal work by Stanley Miller on the abiogenesis of amino acids \cite{miller_production_1953}, ~~the~~ so-called prebiotic chemistry has reached important achievements (reviewed by Ruiz-Mirazo \textit{et al}. \cite{ruiz-mirazo_prebiotic_2014}). Prebiotic chemistry focuses on revealing how some organics of biological relevance can be formed via plausible chemical routes from simple primitive molecules. Some of these available molecules, once formed, could have acted (\textit{i}) as catalysts and/or building blocks in early metabolic cycles, and (\textit{ii}) as catalysts for the synthesis of large chain-like molecules, i.e., proteins and

nucleic acids. For the origin of life, ~~it is indeed decisive~~ both the onset of reaction networks, and the transition from small, low molecular weight compounds to macromolecules is decisive.

As we have recently focused our attention on the role of peptides in these prebiotic scenarios \cite{gorlero_ser-his_2009,wieczorek_formation_2013,adamala_open_2014}, here we aim to review some experimental facts on the discovery that a simple dipeptide, Ser-His (Figure \ref{fig:ser-his}), is capable of catalysing the coupling reaction of amino acid derivatives or nucleotide derivatives. Then, starting from this specific evidence, we discuss in more general way what could have been the roles of short and random peptides in origins of life. Screening of peptide libraries, organocatalysis mechanisms, molecular recognition, and interaction with primitive membranes are possible fields of inquiry for further developments of this research.

```
\begin{figure}[H]
\centering
\includegraphics[width=7.5cm]{Ser-His}
\caption{Chemical structure of Ser-His with evidence on its several functional groups. Imidazole ring is shown in its neutral \textit{N}$^{\epsilon}$-H tautomeric form.}
\label{fig:ser-his}
\end{figure}
```

The paper is organized in five sections. Firstly, we introduce organocatalysis, as it is known today in organic chemistry (Section \ref{sect:what_is_organocatalysis}). It is highly remarkable that one of the most modern and cutting-edge chemical approaches, often used to synthesize complex molecules with high chemo-, regio-, and stereoselectivity, relies on the same mechanisms that probably contributed to the emergence of biomolecules and cellular life billions years ago. After a quickly review on the current ideas about the prebiotic origin of amino acids and peptides (Section \ref{sect:origin_peptides}), ~~it follows~~ a detailed discussion of the two important synthetic reactions catalysed by Ser-His, namely the formation of peptide bonds and phosphodiester bonds (Section \ref{sect:ser-his}) follows. This part is enriched by a discussion on computer-generated three-dimensional models of Ser-His and its isomer His-Ser (not functioning as catalyst). The fragment condensation model is then presented and discussed, by referring to the potential role of random peptides with ligation activity (Section \ref{sect:fragment_condensation}). Finally, some recent accounts about other potential roles of peptides in origin of life are presented (Section \ref{sect:other}).

It is noteworthy that the main finding discussed in this review stems from a fundamental principle in chemistry, ~~the~~ microscopic reversibility. A catalyst of the reaction \ce{A\bond{-}B -> A + B}

also catalyses the reverse reaction \ce{A + B -> A\bond{-}B} with the same (reversed) mechanism, but it cannot change the DG of the reaction. Intrigued by a YuFen Zhao work reporting that Ser-His (and other short peptides as Cys-His, Ser-His-Gly, Ser-His-His, Ser-His-Asp, Ser-Gly-His-His, Ser-Gly-Gly-His-His) is capable of cleaving peptide and phosphodiester bonds \cite{li_dipeptide_2000}, we reasoned that Ser-His should be also able to catalyse the reverse reaction. The interesting fact is that these reverse reactions are exactly one of the holy grails of prebiotic chemistry, those reactions that lead to the formation of peptide and phosphodiester bonds, thus potentially leading to long peptides and long nucleic acids. As it is well known, peptide synthesis has been indeed accomplished by proteases with the caveat of being able to shift the thermodynamical equilibrium toward the desired products or by using activated acyl donors \cite{chaiken_use_1982,fruton_proteinase-catalyzed_1982,jakubke_basic_1985}.

\section{What is organocatalysis? The organic chemist’s view.}

\label{sect:what_is_organocatalysis}

Although specific examples of organic catalysts have been known ~~since~~ for a long time, the modern term `\textit{organocatalysis}' and its field of application have emerged only recently in synthetic chemistry, as discussed in an interesting essay \cite{macmillan_advent_2008}. Prebiotic chemistry, which is somehow a niche branch of chemistry, unfortunately did not contribute to the conceptualisation of organocatalysis. Paradoxically, however, it is prebiotic chemistry the field where organocatalysis is such a native and powerful concept that surely will be determinant for explaining age-long unsolved questions such as the origin of macromolecules, of metabolic cycles (see Smith and Morowitz contribution in \cite{multiple_authors_workshop_2010}), the importance of membrane-related processes and many other primitive mechanisms.

Organocatalysis can be defined as the use of small, low-molecular weight organic compounds, predominately composed of carbon, hydrogen, oxygen, sulphur and phosphorous to accelerate chemical reactions. In the field of organic synthesis, the use of organocatalyst date back to Liebig’s preparation of oxamide from dicyano and water (1860), and through the years the number of examples highlighting the application of such a strategy has become countless. However the real renaissance of organocatalysis in the organic chemistry practice should be established in 2000 with the List’s breakthrough concerning the first proline-catalyzed direct asymmetric aldol reaction (yield 97\%, enantiomeric excess 96\%) \cite{list_proline-catalyzed_2000}, and by the first report on an enantioselective organocatalytic Diels-Alder reaction \cite{ahrendt_new_2000}. This work contributed to the emergence of organocatalysis by showing that small organic molecules (such as proline) could catalyze the same chemical reactions as much larger organic molecules (enzymes) do in biochemistry, and following similar mechanisms \cite{macmillan_advent_2008} (note, however, that organocatalysis is not necessarily `biomimetic').

A growing amount of publications appeared later, and asymmetric organocatalysis has been designated as the third general approach to the catalytic production of enantiomerically pure compounds \cite{macmillan_advent_2008}. Indeed, undisputed masters in organic chemistry as well as a growing community of research groups have devoted their efforts toward the development of novel organocatalytic strategies that offer a valuable effective alternative to the well-established transition metal and enzymatic methods. In organic synthesis, the organocatalytic approach

provides several advantages: (\textit{i}) usually, the catalysts are quite cheaper than the transition metal complexes, readily available, more stable and easier to be synthesized, (\textit{ii}) the reactions can be generally performed under low demanding conditions without the regular need for inert atmosphere or dry solvents, and most important, (\textit{iii}) different activation modes are possible with respect ~~of~~ to substrates, reagents and reactions.

Within this context, one of the biggest attractiveness of organocatalysts relies on their abilities of variously interacting with considerably different substrates according to the accurate choice of their chemical structure and properties. Both covalent and non-covalent interactions and activation of substrates are in the realm of organocatalysis, very often in chemio-, regio-, and stereoselective manner. Moreover, dual and cooperative catalysis, which allows the catalyst to bind to more than one substrate by using different activation modes, has paved the way to original reactions giving access to novel useful scaffolds. Clearly, it is expected that a larger variety of organocatalysts and modes of action are going to be developed in future. The common feature, however, will be the same: conditioning the course of a reaction by close interaction with the reaction partners, in agreement to a minimalistic version of the lock-and-key paradigm of enzyme action \cite{duncan_short_2015}.

As this paper is devoted to organocatalysis in primitive chemical systems, ~~the~~ readers interested in organocatalysts for modern chemical synthesis should refer to reviews and books focusing on that subject. Just to enumerate some examples, amino acids and short peptides have been used as catalysts for the following reactions: Robinson annulation, intermolecular aldol reaction, Mannich reaction, Michael addition, hydrocyanation of aldehydes and imines, epoxidation, acyl transfer reactions, conjugate additions of thiols and azides, cycloadditions, phosphorylation, and a series of domino as well as multicomponent reactions \cite{jarvo_amino_2002}.

\section{Origin of peptides}
\label{sect:origin_peptides}
It is commonly accepted that amino acids, at least some of the α-amino acids that we know today, are easily formed abiotically. Amino acids were therefore generally available, together with a plethora of other small organic compounds that were similarly synthesized by `natural' processes. The famous Miller-Urey experiment, for example, produced a mixture of simple amino acids \cite{miller_production_1953}. Moreover, amino acids have been also found in meteorites \cite{pizzarello_chemistry_2006}. A consensus list of prebiotic amino acids includes Ala, Asp, Glu, Gly, Ile, Leu, Pro, Ser, Thr, and Val \cite{doi_high_2005,zaia_which_2008,mcdonald_biochemical_2010,longo_protein_2012}, whose mechanisms of formation follow a Strecker synthesis model \cite{wolman_nonprotein_1972,lerner_strecker_1993,huber_alpha-hydroxy_2006,longo_simplified_2013}.

From the point of view of the discussion in this paper we are most interested in the prebiotic availability of aspartate, serine and histidine. The first two of those amino acids form easily in simulated prebiotic conditions and have been reported in several works. Histidine, however, is not encountered as a straightforward product in, for example, Strecker-like synthesis. It has been noted however (Shen, C., Yang, L., Miller, S. L., & Oró, J. (1987). Prebiotic synthesis of imidazole-4-acetaldehyde and histidine. *Origins of Life and Evolution of Biospheres*, *17*(3), 295-305.), that once imidazole can be obtained in a prebiotic environment, it should be, in principle, possible to get to histidine through the Strecker synthesis. Prebiotic imidazole synthesis have been reported in several set-ups (Oró, J., Basile, B., Cortes, S., Shen, C., & Yamrom, T. (1984). The prebiotic synthesis and catalytic role of imidazoles and other condensing agents. *Origins of Life and Evolution of Biospheres*, *14*(1), 237-242.; Ferris, J. P., & Orgel, L. E. (1966). Studies in Prebiotic Synthesis. I. Aminomalononitrile and 4-Amino-5-cyanoimidazole1, 2. *Journal of the American Chemical Society*, *88*(16), 3829-3831.; Vázquez-Salazar, A., Tan, G., Stockton, A., Fani, R., Becerra, A., & Lazcano, A. (2016). Can an Imidazole Be Formed from an Alanyl-Seryl-Glycine Tripeptide under Possible Prebiotic Conditions?. *Origins of Life and Evolution of Biospheres*, 1-10.). Basing on the premise of Strecker process trough imidazole intermediate the prebiotic synthesis of histidine has been reported starting from erythrose and formamide (Shen, C., Yang, L., Miller, S. L., & Oró, J. (1990). Prebiotic synthesis of histidine. *Journal of molecular evolution*, *31*(3), 167-174.). Some have argued that such process does not fulfill the criteria to be considered prebiotic (Vázquez-

Salazar, A., Tan, G., Stockton, A., Fani, R., Becerra, A., & Lazcano, A. (2016). Can an Imidazole Be Formed from an Alanyl-Seryl-Glycine Tripeptide under Possible Prebiotic Conditions?. *Origins of Life and Evolution of Biospheres*, 1-10.) and therefore wheter this amino acid could in fact be available in early stages of prebiotic evolution remains open for debate.

Although the origin of some amino acids is not perceived as problematic (and it has been demonstrated that some of them may also act as organocatalysts \cite{pizzarello_prebiotic_2004,zou_small_2005}), their oligomerization to form early peptides, is a more intriguing process. The endoergonic standard Gibbs free energy change associated to the formation of peptide bonds in aqueous solutions from carboxylate and ammonium ions is about +10-20 kJ/mol \cite{martin_free_1998}, implying that the reaction is not thermodynamically favored. This simple consideration explains why the investigations on primitive condensation mechanisms leading to polypeptides are intriguing and challenging. Making peptide bonds plausibly required a combination of activation pathways in order to overcome and bypass this thermodynamic barrier. It is indeed remarkable that the formation of peptide bonds in the ribosome does not occur by direct condensation of two amino acid moieties. An `activated' carboxylic group is employed, namely the aminoacyl-tRNA adduct, which is an amino acid ester, whose formation ultimately requires ATP energy. In biochemistry, the peptide bonds are formed via ester aminolysis, not by carboxylic acid/amine condensation.

But the chemical path to join amino acids is not the only problem in the origin of peptides. Two others relevant issues are their chirality (were early peptides homochiral?), and the peptide sequence (did specific amino acid sequences emerged? And according to which mechanism?).

Coming back to the chemical aspects of amino acids oligomerization, four main routes are classically discussed: the thermal condensation in dry state or in liquid phase, the removal of water in dry/wet cycles, the salt-induced condensation in aqueous solution (catalyzed by cupric ions), and the oligomerization of activated amino acids (in form of \textit{N}-carboxyanhydrides, thioesters, and other forms). In many cases, solid surfaces like clay and other minerals improve the yield and/or favor homochirality; conversely not much studies have been devoted to the peptide sequences. A detailed review on the principal results in the field has been recently published \cite{ruiz-mirazo_prebiotic_2014}. Here we give just some examples of prebiotic routes to oligopeptides.

It was proposed that racemic mixtures of amino acid alkyl and thio-alkyl esters self-assembled on the water/air interface can undergo spontaneous condensation, forming randomly-sequenced peptide chains \cite{zepik_chiral_2002}. However, recent studies show that in case of short side chain natural amino acids, the reaction does not proceed beyond the stage of di- and tripeptides \cite{eliash_structure_2004}, so it cannot be regarded as a prebiotic way of obtaining long and enantiomerically-pure peptides. Formation of peptides can be induced by high salt environment \cite{suwannachot_mutual_1999}, and under certain conditions high enantioselectivity of the oligomerisation of amino acids was observed \cite{li_isoleucine_2013}. It has been recently suggested that is possible to envision prebiotically plausible mechanism of stereoselective peptide bond formation based on glutathione synthesis \cite{fiser_glutathione_2015}. Amino acids and other simple carboxylic acids can be synthesized in iron-catalyzed redox reactions \cite{schoonen_energetics_1999}. Cyanine-rich reducing environment has been shown to yield arginine – an amino acid involved in many peptide interactions with nucleic acids \cite{patel_common_2015}, recently it has been also demonstrated that peptidyl-RNA compexes could form under conditions simulating prebiotic RNA oligomerization and that such hybrid molecules could extend RNA's stability, thus hinting at early reasons for RNA-peptide interactions which evntualy led to translation system of modern life (Jauker, M., Griesser, H., & Richert, C. (2015). Spontaneous formation of RNA strands, peptidyl RNA, and cofactors. Angewandte Chemie International Edition, 54(48), 14564-14569.).

A more general, prebiotically plausible peptide synthesis mechanism was proposed, involving 2-thiono-5-oxazolidones generated in reactions of amino acids with carbon disulfide \cite{leman_peptide_2015} or \textit{N}-carboxyanhydrides (Leuchs anhydrides) \cite{ehler_nn-carbonyldiimidazole-induced_1976,taillades_n-carbamoyl_1999,danger_peptide_2006} from carbonyl sulfide \cite{leman_carbonyl_2004}. Interestingly, liposome compartments can assist sequence-specific, stereoselective peptide formation on the surface of the bilayer \cite{hitz_liposome-assisted_2000,hitz_stereoselectivity_2001}, similar behaviour has been recently reported in regard to vesicles buil~~d~~t from more prebiotically plausible simple fatty acids (Murillo-Sánchez, S., Beaufils, D., Mañas, J. M. G., Pascal, R., & Ruiz-Mirazo, K. (2016). Fatty acids' double role in the prebiotic formation of a hydrophobic dipeptide. *Chemical Science*, *7*(5), 3406-3413.).

These and many other results show that the presence of small peptides in early Earth was quite plausible. In such a huge combinatorial peptide library, highly diverse ~~with respect to~~in length,

chirality and sequence, several interesting compounds might have been originated and reciprocal interaction could start. Theoretical accounts for chemical networks can be found in the work of Kauffman \cite{kauffman_origins_1993}, although it should be stressed that most of the mechanisms discussed involve demonstrated formation of ~~relativly~~ relatively short oligopeptides – dimers and trimers. More attention should be given ~~at~~to demonstrating the formation of longer chains, for example decapeptides, which would be more appropriate for Kauffman's scenario. Some of these primitive peptides could have simply degraded, or further elongated, or – more excitingly – unveil catalytic properties. Short random peptides could have been formed spontaneously by the above-mentioned mechanisms and thus help ~~in decisive way~~ the onset of protobiological mechanisms (like autocatalytic and cross-catalytic cycles, complex formation with metals or other non-peptide molecules, membrane functionalization and control of trans-membrane traffic, new peptide formation by chain elongation or fragment condensation, synthesis and stabilization of nucleic acids, and so on)~~.~~ in significant ways.

There is a prolific field of study involving self-replicating networks of helical peptide replicators as demonstrated in the labs of Ghadiri (Saghatelian, A., Yokobayashi, Y., Soltani, K., & Ghadiri, M. R. (2001). A chiroselective peptide replicator. *Nature*, *409*(6822), 797-801, Lee, D. H., Severin, K., and Ghadiri, M. R. "Autocatalytic networks: the transition from molecular self-replication to molecular ecosystems." Curr. Opin. Chem. Biol. 1997, 1, 491-496.), Chmielewski (Issac, R., Ham, Y. W., & Chmielewski, J. (2001). The design of self-replicating helical peptides. *Current opinion in structural biology*, *11*(4), 458-463, A pH-modulated, self-replicating peptide By: Yao, S; Ghosh, I; Zutshi, R; et al. JOURNAL OF THE AMERICAN CHEMICAL SOCIETY Volume: 119 Issue: 43 Pages: 10559-10560~~.~~) and Ashkenasy (Tena□Solsona, M., Nanda, J., Díaz□Oltra, S., Chotera, A., Ashkenasy, G., & Escuder, B. (2016). Emergent Catalytic Behavior of Self□Assembled Low Molecular Weight Peptide□Based Aggregates and Hydrogels. *Chemistry–A European Journal*, *22*(19), 6687-6694., B. Rubinov, N. Wagner, H. Rapaport, G. Ashkenasy, "Self Replicating Amphiphilic β-Sheet Peptides", Angew. Chem. 2009, 121, 6811-6814.). ~~Although, otherwise very~~In these interesting reports, it is shown that ~~those works in which~~ some specific α-helical or β-sheet peptides (i.e., with specific sequences) can undergo to a self-replicative process, in ~~work in~~ a manner that is reminiscent, conceptually,~~more familiar~~ to nucleic acids template-based replication. Although these works might be relevant in the broader origin of life field in discussion on the emergence of mutually catalytic networks (and propagation of homochirality), a full discussion of this chemistry lies outside of the scope of this review.~~ do not seem to have a relevance forbe relevent to the discussion of prebiotic peptide formation. A, and as such will not be discussed~~

~~in this work. Although, these works might be relevant in the broader origin of life field in discussion on the emergence of mutually catalytic networks.~~

Envisioning a primitive scenario with early peptide catalysts is fascinating, but ~~to look~~looking for a needle in a haystack of all possible sequences is not easy. However such studies may lead to new chemistries with great explanatory value for the origins of life. In some cases, such peptide catalysts could alleviate the thermodynamic issues concerning ~~the~~ condensation reactions (requiring a way to shift the equilibrium toward the products, via the removal of products or via activation of the substrates), or give a hint to understanding early protocells. As it will be discussed in the next section, Ser-His is probably the shortest peptide that has shown a surprising catalytic capacity.

\section{Seryl-Histidine (Ser-His) catalyzes the formation of peptide bonds and phosphodiester bonds}
\label{sect:ser-his}

\subsection{The discovery of Ser-His hydrolase activity}
\label{subsect:discovery}
From an origin-of-life perspective, a very intriguing report is probably the discovery of seryl-histidine (Ser-His) and seryl-histidyl-aspartate (Ser-His-Asp) hydrolytic activity. Ser-His and Ser-His-Asp could hydrolyse esters as well as proteins and nucleic acids \cite{li_dipeptide_2000} (but see also the note at the end of the text). There are many specialized enzymes catalysing those reactions. Most of them are serine hydrolases. Ser/His/Asp is the most common catalytic triad known from enzymatic studies \cite{berg_biochemistry_2002}. It is to be found in many important enzymes, for example chymotrypsin, subtilisin or phospholipases A2. The principle behind its action has been independently invented in the course of evolution at least 23 times \cite{buller_intrinsic_2013}. Serine hydrolases comprise approximately 1\% of the genes in the human proteome \cite{simon_activity-based_2010}.

The mechanism of the serine triad catalysis is one of the best understood among all enzymatic activities (Figure \ref{fig:triad}). The three groups act as a nucleophile (serine hydroxyl), base catalyst (histidine imidazole ring) and an orienting/stabilizing group (aspartate) \cite{berg_biochemistry_2002}. Histidine is bound and stabilised by aspartate in such a way that it ~~is polarising~~ polarizes the nucleophile thus allowing it to successfully perform nucleophilic attack on the substrate (usually the carbonyl carbon of a peptide). Carbonyl oxygen accepts an electron and a tetrahedral intermediate stabilised by oxyanion hole is form. The electrons from carbonyl oxygen collapse back to the carbonyl carbon ejecting half of the substrate. The other half of the substrate is still bound to the enzyme forming the acyl-enzyme intermediate. The acyl-enzyme intermediate is then attacked by another nucleophile from the environment. Briefly, another tetrahedral intermediate is formed, which then ejects the enzyme while releasing the product. If the new nucleophile is water the result is hydrolysis, if it is another organic substrate the result is acyl transfer.

```
\begin{figure}[H]
\centering
\includegraphics[width=14cm]{triade}
```

\caption{Mechanism of serine protease. The catalytic triad Ser/His/Asp acts in concerted manner and cleaves the peptide bond in two steps. The acyl group is firstly transferred to Ser hydroxyl oxygen, then to water. See details in the text. The dashed lines indicate favourable interactions between the negatively charged aspartate residue and the positively charged histidine residue, which make the histidine residue a more powerful base. Note the `oxyanion hole' which stabilizes via hydrogen bonding with amide N-H the negative oxygen of the tetrahedral intermediate \cite{berg_biochemistry_2002}.}
\label{fig:triad}
\end{figure}

The structure and activity of Ser-His peptide, as well as its corresponding tripeptide Ser-His-Asp, has prompted speculations about the mechanism of its catalytic properties. Being a minimalistic analogue of serine hydrolases has been the most obvious postulate \cite{li_dipeptide_2000}. The group of YuFen Zhao, who first reported on the Ser-His activity has followed with a number of studies on hydrolytic activity, analytics and modelling of this interesting dipeptide \cite{chen_cleavage_2000,li_seryl-histidine_2002,du_appraisal_2002,sun_molecular_2003,sun_molecular_2004-1, sun_molecular_2004,zeng_study_2005,ma_dna_2008,liu_structural_2009,liu_evaluation_2010}. However, none of those studies have actually attempted to elucidate in details the mechanism by which Ser-His hydrolysis functions.

As it often happens in research, Ser-His was discovered by serendipity. It is interesting to report here the words of Zhao and collaborators revealing how Ser-His activity was noticed:
\begin{quotation}
``In the studies of the interaction between \textit{N}-phosphoamino acids with DNA, it was found that the aged solution of \textit{N}-phosphorus serine in a saturated histidine buffer exhibited DNA cleavage activity, not the fresh one. Finally, it was clarified that the seryl-histidine dipeptide which formed in the solution was responsible for the DNA cleavage (Ma, 1996)" \cite{ma_dna_2008}.
\end{quotation}

\subsection{Peptide bond formation}
\label{subsect:peptide_bond_formation}

From a prebiotic perspective, hydrolysis (proteolysis) is not a desired catalytic activity. Synthesis (condensation, reverse proteolysis) is rather preferred, so that the reactions could drive formation of ever more complex molecules. However, as we have anticipated, hydrolysis is thermodynamically

favored in aqueous environment. Following the discussion presented by several authors \cite{jakubke_basic_1985,martin_free_1998,bongers_enzymatic_1994}, the condensation reaction between two charged amino acids (or peptides) in water is composed by two elementary steps, namely, a proton exchange process and an aminolysis reaction, defined in Figure \ref{fig:condensation}a ($\Delta G^{\circ}_{cond} = \Delta G^{\circ}_{exch} + \Delta G^{\circ}_{am} $). It follows that the condensation equilibrium constant (K_{cond}) can be written as the product between the proton exchange constant (K_{exch}) and the aminolysis constant (K_{am}). K_{exch} is the ratio between the acidity constants of free amino acid terminal ammonium ion (10^{-8} — 10^{-9}) and carboxylic acid (10^{-2} — 10^{-3}).

\begin{figure}[H]
\centering
\includegraphics[width=8cm]{amminolisi}
\caption{Formation of peptidic amide bond. (\textbf{a}) Condensation of free amino acids in water, where the reacting groups (terminal carboxylate and terminal ammonium) are charged and unreactive. A proton transfer from the ammonium group to carboxylate would lead to a reactive couple undergoing aminolysis. But actually K_{exch} favors the charged compounds. As K_{cond} = K_{exch} K_{am}, the main thermodynamic barrier to amino acid condensation in water is their presence as charged state, whereas K_{am} would be in favor of the amide. (\textbf{b}) Aminolysis of activated acyl derivative. In contrary to free amino acid condensation, ester aminolysis is thermodynamically favored (typically, K'_{am} 50-60), but the reaction pathway may be hindered by a kinetic barrier, and if carried out in water, hydrolysis competes with aminolysis.}
\label{fig:condensation}
\end{figure}

\begin{align}
K_{exch} &= \frac{[\ce{RCOOH}][\ce{R'NH2}]}{[\ce{RCOO-}][\ce{R'NH3+}]} = \frac{K_{a,\ce{R'NH3+}}}{K_{a,\ce{RCOOH}}} \\
K_{am} &= \frac{[\ce{RCONHR'}][\ce{H2O}]}{[\ce{RCOOH}][\ce{R'NH2}]}\\
K_{cond} = &= \frac{[\ce{RCONHR'}][\ce{H2O}]}{[\ce{RCOO-}][\ce{R'NH3+}]} = K_{exch} K_{am}\\
K_{cond} &= \frac{K_{a,\ce{R'NH3+}}}{K_{a,\ce{RCOOH}}} K_{am}
\end{align}

The overall free energy change is positive (a typical value of ΔG°_{cond} is ca. +20 kJ/mol, or K_{cond} ca. 3 $\times$ 10^{-4}). It is the charged nature of the reactants in water

the thermodynamic reason for making the free amino acids condensation an endoergonic reaction. Work should be done to remove the charge from the carboxylate and ammonium ions in water (this depends on the pK_a values of the reacting groups, but typically ΔG°_{exch} ca. +30 kJ/mol, K_{exch} ca. 4 $\times$ 10^{-6}). It results that the pure aminolysis reaction (\ce{RCOOH + R'NH2}) is per se not unfavored (ΔG°_{am} ca. –10 kJ/mol; K_{am} ca. 60, as in the case of ester aminolysis, see below).

The direction of the condensation reaction can be forced to the right~~left~~ (toward the condensation product) ~~responding~~ using ~~to~~ the ratio between the chemical species (thermodynamic control) \cite{schellenberger_protease-catalyzed_1991}. In water-rich environments hydrolysis prevails, in water-poor environments condensation ~~will become~~ becomes more favorable. Using a large molar excess of the reactants, or removal one of the products by precipitation, evaporation, specific complexation, phase separation, migration to another phase, are the tactics for carrying out the condensation reaction against the unfavourable ΔG°_{cond}.

A second possibility to circumvent the hostile thermodynamics of peptide bond formation from free amino acids focuses on the use of `activated' substrates (i.e., better acyl donors, such as carboxylic acid esters or similar compounds) (Figure \ref{fig:condensation}b). Ribosomes catalyze the peptide bond formation in this way. The aminolysis reaction of an ester is thermodynamically favored (at pH 7, typical ΔG°' ca. –10 kJ/mol, deduced from thermodynamic values \cite{jencks_free_1976}), due to the greatest bonds strength and stability of amides when compared to esters, but catalysts are nevertheless important to overcome the kinetic barrier typically involved in the breakdown of the tetrahedral intermediate. This second strategy (kinetic control) \cite{schellenberger_protease-catalyzed_1991} has been exploited in the applications of enzymes in organic synthesis \cite{schuster_enzyme-catalyzed_1990} \cite{bordusa_proteases_2002}.

With these notions in mind, and inspired by the principle of microscopic reversibility, we ~~started~~ began investigations ~~on~~ into the possibility of using Ser-His as a catalyst of peptide bond formation \cite{gorlero_ser-his_2009}. In particular, we have combined the above-mentioned strategies, i.e., activation of the substrate and removal of the product. We have demonstrated that Ser-His catalyzes peptide bond formation between a \textit{C}-terminus of an activated amino acid (\textit{N}-protected) and the free \textit{N}-terminus of another amino acid (\textit{C}-protected). The activating group on the \textit{C}-terminus is a carboxylic acid methyl or ethyl ester (Figure \ref{fig:macha}). It ~~has to~~ should be noted that these reactants should be intended as models of

origin of life-relevant activated substrates. Interestingly, whereas Ser-His is capable of catalyzing the coupling reaction, His-Ser is not, and neither ~~is~~are serine or histidine alone or mixed together. The same phenomenon has been observed in the hydrolysis reactions \cite{li_dipeptide_2000}. It seems that reversing the order of amino acids changes the three dimensional arrangements of side chains as well as their geometrical relation (Figure \ref{fig:ser-his}). These structural changes translate in a mismatching arrangement of functional groups necessary to perform catalysis.

The reaction most extensively studied has been condensation between two amino acid substrates (``1+1" combination, to give a dipeptide): \textit{N}-acetylated phenylalanine ethyl ester and leucine amide (Figure \ref{fig:macha}), that lead to the water-insoluble dipeptide \textit{N}-acetyl phenylalanyl-leucinamide \cite{gorlero_ser-his_2009}. The reaction has almost no uncatalyzed background and proceeds rapidly (within minutes) by α-chymotrypsin catalysis. The phenyl ring ensures easy detection by UV absorbance. Usually HPLC analysis is employed to monitor the reaction. In presence of Ser-His, ~~T~~the reaction proceeds better at neutral-slightly alkaline pH (7-9); higher pH values favor the substrate hydrolysis. The latter is reduced by working at low temperature (5-15 $^{\circ}$C), whereas it prevails at higher temperatures. Under optimized conditions, yields go from 20\% to 50\%, mainly depending on the duration of reaction (up to 30 days).

```
\begin{figure}[H]
\centering
\includegraphics[width=12cm]{macha}
\caption{Ser-His catalyzed peptide formation \cite{gorlero_ser-his_2009}. \textit{N}-protected and
\textit{C}-activated phenylalanine residue (AcPheOEt) reacts with \textit{C}-protected leucine
residue (HLeuNH$_2$) to give ethanol and the desired dipeptide (AcPheLeuNH$_2$) in 20-50\%
yield (depending on incubation time, up to 30 days), at pH 7 to 9 (Britton-Robinson buffer was
used, including acetate, phosphate and borate). The reaction has been tested from 4 to 65
$^{\circ}$C, and runs well also in the presence of cupric ions and urea. Note that
AcPheLeuNH$_2$ precipitates in the reaction conditions, shifting the equilibrium to the right. As
comparison, if $\alpha$-chymotrypsin is used instead of Ser-His, the reaction occurs in few
minutes. Together with aminolysis, AcPheOEt hydrolysis is observed (AcPheOH).}
\label{fig:macha}
\end{figure}
```

Higher peptides were formed analogously. In addition to the ``1+1" combination, other peptides were synthesized according to the ``1+2", ``2+1" and ``2+2" combinations, to give, respectively, tri- and tetrapeptide (Figure \ref{fig:all_reactions}). These results illustrate the applicability of Ser-His as potential peptide ligase of peptide fragments, to form longer peptides. What ~~is~~ 's more, the reaction was extended to peptide nucleic acid (PNA) monomer substrates, producing, in a single pot reaction, PNA-dimer, trimer and tetramer \cite{gorlero_ser-his_2009} (Figure 5, last line). In

contrary to the other cases, the latter reaction proceeds by joining `bifunctional' substrates, namely having an electrophile and a nucleophile moiet~~ies~~y in the same molecule, so that the oligomerization is possible. Ser-His, therefore, can promote also elongation reactions by repetitive addition of monomers to a growing chain through ~~a~~ formation of amide bonds, which in principle should be easily extenda~~v~~ble towards growing peptide chains.

```
\begin{figure}[H]
\centering
\includegraphics[width=12cm]{combinazioni}
\caption{Schematic representation of the peptide-forming reaction catalyzed by Ser-His
\cite{gorlero_ser-his_2009}. In addition to the ``1+1" case, yielding a dipeptide, other combinations
have been explored, up to the formation of tetrapeptide by a ``2+2" condensation scheme.
Moreover, by using a ``bifunctional" substrates, namely peptides having both an electrophile moiety
and a nucleophile counterpart in the same molecules, it was shown the oligomerization of peptides
(H-Trp-OEt and H-Phe-OEt, up to the dimer, \textit{n} = 2) and peptide nucleic acids (PNAs) units
(up to a tetramer, \textit{n} = 4).}
\label{fig:all_reactions}
\end{figure}
```

Prebiotically plausible conditions are usually understood as water environments in mild temperature regimes \cite{orgel_prebiotic_2004}. In such conditions, hydrolysis of the activated substrate is favoured. This was indeed also observed under Ser-His catalysis. Thus, the two reactions (peptide bond formation and hydrolysis) are in competition with each other, as it happens in kinetically controlled peptide synthesis \cite{schellenberger_protease-catalyzed_1991}. Usually a large part of the acyl substrate (up to 50\%) ends up as a free acid compound. The available data do not help for the determination of the mechanisms underlying coupling and hydrolysis reactions, but the presence of a covalent complex between Ser-His and the acyl substrate is a possibility under discussion. Small amount of a compound with unknown structure and a molecular mass corresponding to covalent adducts have been isolated (Gorlero \textit{et al}., unpublished results) when H-Phe-OEt or H-Trp-OEt was incubated with Ser-His attempting to synthesize Trp or Phe oligomers (conditions of Figure \ref{fig:all_reactions}, last line~~see Figure \ref{fig:all_reactions}~~).

Under the explored experimental conditions, for the transfer reaction to occur we have to ensure that the product of condensation has low solubility in water. This characteristic ensures the forming product will drop out from the solution and form insoluble precipitate, thus driving the equilibrium of the reaction towards formation of peptide bond even in an aqueous environment (Figure \ref{fig:macha}). Therefore the established method allows for the synthesis of hydrophobic peptides, with the general rule being that the product needs to be more hydrophobic than either of

the substrates. The bigger the difference in partition-coefficient between the substrates and the product, the more efficient is the synthesis. (\textit{Nota bene}. One of the consequences of this fact is that although we could have a case of peptide catalysed peptide synthesis -- Ser-His that would catalyze its own formation -- there is no straightforward way for an autocatalytic circuit where the catalyst and the product are the same molecular species. The catalyst itself, Ser-His, is extremely polar molecule with four hydrogen donating/accepting functional groups).

The removal of the product from the reaction mixture does not necessarily mean precipitation. It can also mean a migration of the product to a different phase. If a reaction Ac-Phe-OEt + H-Leu-NH$_2$ is carried out in a two-phase system, for example in the presence of lipid vesicles -— the interior of their bilayers will be the hydrophobic environment to which water-insoluble dipeptide can migrate \cite{adamala_competition_2013}. The accumulation of the dipeptide in the membrane has been shown to change the stability of the membrane. This can lead to a mechanism of competitive growth of protocell liposomes containing the catalyst Ser-His and thus the produced dipeptide over those that do not posses it. This neat situation has been heralded as an example of selective processes that could operate even before the invention of genetic material in the population of prebiotic vesicles \cite{adamala_competition_2013}. It is an example of something that has been dubbed `supramolecular selection' -— a process that operates in the prebiotic ecology stage of prebiotic evolution and ensures selection of certain macromolecular assemblies over others \cite{wieczorek_prebiotic_2012}.
Finally, it should be mentioned that in another study, Ser-His was employed to promote peptide bond formation between two groups linked to ends of a synthetic PNA stem loop structure \cite{singhal_toward_2014}, but in this case its effectiveness as an organocatalyst was poor.

\subsection{Phosphodiester bond formation}
\label{subsect:phosphodiester_bond_formation}
Encouraged by the promising results of Ser-His catalysed peptide synthesis we asked a question whether similar reversal of hydrolysis could be obtained with nucleic acids. Our campaign explored several different paths. We achieved success with an approach involving polymerization of imidazole-activated mononucleotides in water/ice eutectic mixture where water is cooled below its freezing point but above the eutectic point \cite{wieczorek_formation_2013}. Under such conditions most of the water is sequesteredtrapped in the form of ice crystals and the rest of the

solutes is up-concentrated in the remaining liquid microchannels. This strategy achieves two functions: first, it ~~is radically lowering~~radically lowers water activity as most of it is in the solid state and does not participate in the reaction; second,~~ the~~ previously diluted reactants ~~are reaching~~ reach concentrations in which their interactions are facilitated. Another advantage is that at such low temperatures, (ca. –18 $^{\circ}$C) fragile imidazole-activated nucleotides have prolonged half-life \cite{kanavarioti_eutectic_2001}. The imidazole activation method is widely used as a model for non-enzymatic oligomerization \cite{orgel_prebiotic_2004,monnard_eutectic_2008}.

Although the achieved length of RNA oligomers has been limited to ~~only~~ only a few nucleotides, the catalytic formation of phosphodiester bond by Ser-His has been well studied and the proposed mechanism of the catalysis has been ~~published~~ reported thanks to the isolation of an intermediate complex \cite{wieczorek_formation_2013,adamala_open_2014} (Figure \ref{fig:rafal}).

```
\begin{figure}[H]
\centering
\includegraphics[width=15cm]{rafal}
\caption{Proposed reaction scheme. Top: The oligomerisation of imidazole-activated nucleotides
\textbf{1} by Ser-His \textbf{2} involves the creation of a covalent linkage between dipeptide and
mononucleotide (\textbf{3}), thereby releasing imidazole (\textbf{4}). Compound \textbf{3}
rapidly forms and is then slowly consumed. It can be attacked by 3'-or 2'-hydroxyl group of another
nucleotide, thereby forming dimers \textbf{9} and \textbf{6} (only 3'–5' bonds shown). Bottom:
alternatively, it can be converted into side products: pyrophosphate dimer \textbf{7}, ribonucleotide
\textbf{5} and an inactive, stable complex \textbf{8} are formed upon nucleophilic attack by a 5'-
phosphate of \textbf{5}, water or the \textit{N}-terminus of dipeptide \textbf{2}, respectively.
Reproduced from Wieczorek \textit{et al}. \cite{wieczorek_formation_2013} with the permission
of Elsevier.}
\label{fig:rafal}
\end{figure}
```

The reaction proceeds much like other RNA polymerizations from imidazol-activated nucleotides. The presence of an imidazole ring covalently attached to 5' phosphorus draws electron cloud~~y~~s away, thus allowing for a partial positive charge to form on the atom. It is subsequently attacked by 2'- or 3'- nucleophilic oxygen from 2'- or 3'-hydroxyl of another nucleotide. Imidazole is released and a phosphodiester bond is formed. The mechanism ~~with the~~ in the presence of Ser-His is very similar. The initial step is a transamination reaction with imidazole being replaced by the Ser-His histidine ring. Because of structural similarity, the Ser-His-capped nucleotide is also an activated form of the nucleotide, like 2-methyl-imidazole or 2-amino-benzimidazole or 1-methyl-adenine

\cite{monnard_eutectic_2008}. The Ser-His-capped intermediate reacts with all nucleophiles present in the system to give, respectively, the desired dinucleotide, a pyrophosphate dimer, and the unactivated nucleotide (Figure \ref{fig:rafal}). It can also react with the free terminal amino group of Ser-His to give a \textit{N}-phosphate peptide-nucleotide adduct. It is unclear whether the reaction of the Ser-His-capped nucleotide with the various nucleophiles proceeds by intramolecular catalysis (e.g., because of specific structural arrangement allowing bound Ser-His to actively take part to the reaction), intermolecular catalysis (e.g., by the involvement of a second Ser-His molecule), or just because the presence of the Ser-His cap as the leaving group is more effective towards polymerization than the bare imidazole.

The involvement of all other Ser-His functional groups (Figure \ref{fig:ser-his}) cannot be excluded. Indeed, Ser-His derivatives such as \textit{N}-acetyl Ser-His and Ser-His amide, as well as the amino acids Ser and His a~~nd~~s well as their mixture do not display any catalysis. This implies that the complete dipeptide structure is important, not just a specific functional group(s). Note, however, that Ala-Ser also catalyzes ~~the~~ phosphodiester bond formation, although with much less efficiency when compared with Ser-His. This ~~evidences~~demonstrates the importance of the serine hydroxy group.

\subsection{A tentative discussion on the catalytic mechanism of Ser-His} \label{subsect:tentative}

Of the two synthetic reactions catalyzed by Ser-His (formation of peptide bonds and phosphodiester bonds) not much is known about their detailed mechanisms. The mechanism emerging from Ser-His catalyzed phosphodiester bond formation Figure \ref{fig:rafal} is quite different from what would be expected (\textit{cf}. Figure \ref{fig:triad}). Here, a covalent intermediate has been isolated ~~where~~with the imidazole ring, rather than the serine hydroxy group. It is again a case of nucleophilic catalysis, but based on Ser-His imidazole, not on the Ser-His hydroxyl group. The essential role of the nucleophilic (unprotonated) imidazole in this mechanism is supported by the fact that the reaction does not occur below pH 6, and that when the Ser-His imidazoyl \textit{N}$^{\epsilon}$ is methylated, the resulting compound does not function as catalyst.

Much less is known about the mechanism of peptide formation (Section \ref{subsect:peptide_bond_formation}). In this case, the pH dependence of ~~the~~ peptide formation

has been determined \cite{gorlero_ser-his_2009} for the system ~~of~~ Figure \ref{fig:macha}. It follows a neat sigmoidal profile with an inflection point at pH 7.85 (Figure \ref{fig:pH}), suggesting that the reaction product is generated by a mechanism where the deprotonation of an acid moiety with ~~such~~ a p\textit{K}$_a$ around such a value is ~~determinant~~fundamental. Although it is not surprising that in many proteins the imidazole p\textit{K}$_a$ can differ (also largely) from the standard value of about 6, depending on the micro-environment \cite{harris_structural_2002}, in this specific case it seems that the protonation status of imidazole of Ser-His does not play a key role. The three Ser-His p\textit{K}$_a$ values are reported to be 2.42 (carboxylic), 6.71 (imidazole), and 7.70 (amino) \cite{leung1999buffered}, and it is known that the p\textit{K}$_a$ of terminal amino group of peptides is around 8. It follows that if the rate-determining step is the initial interaction between Ac-Phe-OEt and Ser-His, the Ser-His terminal amino group might act under the general base catalysis scheme, facilitating the nucleophilic attack of another Ser-His molecule to the ester. If, on the other hand, the rate-determining step is the attack of leucinamide terminal amino group to the carbonyl carbon, the dominant factor~~it~~ is the availability of such unprotonated amino group on the N-end of the $HLeuNH_2$ ~~the dominant factor~~. More detailed studies are needed to clarify these mechan~~istic~~ istic aspects. In any case the mechanism of Ser-His-Asp action is expected to be the same as Ser-His with the carboxyl group of Asp and carboxyl C-end of Ser-His performing similar function in overal mechanism.

```
\begin{figure}[H]
\centering
\includegraphics[width=8cm]{pH}
\caption{Profile of the Ser-His efficiency in promoting the aminolysis reaction shown in Figure
\ref{fig:macha}. The inflection point of the sigmoidal curve, as obtained by non-linear fitting of
experimental data, is at pH 7.85 \cite{gorlero_ser-his_2009}.}
\label{fig:pH}
\end{figure}
```

\subsection{Molecular modelling studies. What can we learn?}

\label{subsect:molecular_modeling}

As we have mentioned, although Ser-His is an organocatalyst for hydrolysis and condensation reactions, it is intriguing that neither the isomer His-Ser, nor the His + Ser mixture have similar activity. To investigate the atomic bases of the different properties of His-Ser and Ser-His dipeptides, molecular dynamics (MD) simulations in explicit solvent were performed at 300 K on

the respective molecular models, considering both tautomeric forms of monoprotonated histidine (protonated either on the \textit{N}$^{\epsilon}$ or on the \textit{N}$^{\delta}$ ring nitrogen). In detail, after energy minimization of the models, the temperature of the system was gradually increased from 0 to 300 K in 100 ps and the systems were simulated for 1 ns at 300 K in the NVT ensemble. The final conformations obtained after 1 ns are shown in Figure \ref{fig:modeling}.

As can be seen from the images, the four models display distinct conformations dictated by their specific stereochemistry. In detail, both the His-Ser and Ser-His dipeptides in the \textit{N}$^{\delta}$-H state (Figure \ref{fig:modeling}a,c) display a weak electrostatic interaction between the \textit{N}-terminal ammonium group and the \textit{C}-terminal carboxylate which is formed very early in the MD simulations. Such an interaction is never observed during the course of the simulations in the \textit{N}$^{\epsilon}$-H dipeptide.
In the case of \textit{N}$^{\epsilon}$-H His-Ser dipeptide (Figure \ref{fig:modeling}b), a strong electrostatic interaction between the dipeptide \textit{N}-terminal ammonium group and the Ser hydroxyl group is formed soon after the beginning of the MD simulations and persists until the end of the simulation time. In principle, in this latter case the observed conformation could lead -- after deprotonation of the terminal \ce{-NH3+}, to polarization of the O-H bond of the Ser hydroxyl group, required to generate the strong nucleophile needed for a serine protease-like catalytic activity (indeed, this is reminiscent of the Lys-Ser catalytic dyad observed for instance in bacterial Type I signal peptidases \cite{ekici_unconventional_2008}). Despite this favourable conformation, experiments show that His-Ser is inactive (pH 10). On the other hand, \textit{N}$^{\delta}$-H Ser-His (Figure \ref{fig:modeling}c) adopts a folded conformation where the Ser hydroxyl group and the His imidazole ring approximately faced ~~to~~ each other, but no specific intramolecular interactions can be recognized from the model. Note that previous work \cite{liu_structural_2009} has shown that \textit{N}$^{\delta}$-H Ser-His has a conformation similar to what has been found by us. Interestingly no evidences for a short and directional intramolecular \ce{O\bond{-}H\bond{....}N} hydrogen bond can be obtained from these 3D-models. The conformation of \textit{N}$^{\epsilon}$-H Ser-His (Figure \ref{fig:modeling}d) resulted to be almost extended.

Therefore, MD simulations results do not provide a straightforward explanation for the occurrence of a serine protease-like catalytic activity in the Ser-His dipeptide as opposed to the His-Ser dipeptide. On the basis of this result, we can put forward the hypothesis that the active species responsible for the Ser-His catalytic properties might not be a monomeric but rather an oligomeric, most likely dimeric, species in which a proper catalytic triad may be formed.

```
\begin{figure}[H]
\centering
\includegraphics[width=12cm]{modeling}
\caption{Molecular models of (\textbf{a}) \textit{N}$^{\delta}$-H His-Ser, (\textbf{b})
\textit{N}$^{\epsilon}$-H His-Ser, (\textbf{c}) \textit{N}$^{\delta}$-H Ser-His and (\textbf{d})
\textit{N}$^{\epsilon}$-H Ser-His, as obtained after 1 ns molecular dynamics simulations in
explicit solvent.}
\label{fig:modeling}
\end{figure}
```

\section{A peptide ``fragment condensation'' scenario}

\label{sect:fragment_condensation}

In Section \ref{sect:origin_peptides} we have seen some of the most common pathways for the origin of peptides in prebiotic conditions. Summarizing, these are: condensation at high temperature or by water removal cycles, salt-induced condensation, and oligomerization of activated monomers. According to this view, a pool of amino acids and oligopeptides of various lengths (typically < 10-mers) and diverse sequences might be considered as the starting point for the emergence of interactive reaction networks, interactions with early membranes, and as feeding material for the synthesis of longer peptides. Organocatalysis helps here.

Some of these spontaneously formed compounds might be endowed ~~of~~ with special structural features, like ~~it happens~~ with Ser-His, and being capable of catalyzing the condensation of the other peptides, according to a fragment condensation pattern --- which is reminiscent of the practice of peptide `chemical ligation' operated by proteases \cite{tan_structural_1995,dawson_synthesis_2000}. Fragment condensation foresees the combination of small peptide oligomers to rapidly converge to quite large chains. Chain elongation, the biochemical way to synthesize biopolymers, is instead based on the growth of a chain by sequential addition, one after the other, of building blocks to one of the two chain extremes. To produce an 80-mer, it needs 79 chain elongation steps; starting from 10-mers, instead, 7 fragment condensation steps are required.

The potential role of fragment condensation in generating long biopolymers has not been widely discussed in the literature, probably because most of the studies focus on mimicking the biological synthesis of biopolymers, that proceeds via template-directed chain elongation. Nucleic acids and protein~~s~~ elongation mechanisms rely on the same recognition strategy, based on nucleotides' complementarity. One could speculate that primitive peptide fragment condensation could be totally random, or guided by molecular recognition (e.g., peptide-peptide or peptide-catalyst). The latter could act as a selection rule in the huge combinatorial space of potentially reacting peptides. Fragment condensation, if played a role in prebiotic times, was functional for endowing the early molecular systems of the first long-enough (and thus likely folded) proteins. Such scenario could anticipate and facilitate the path toward the more sophisticated template-based chain elongation.

Fragment condensation of primitive peptides, unfortunately, lacks of experimental studies, but it is per se an interesting scenario that needs more attention. (Note, however, that a protease-catalyzed oligomerization of a native protein segment in neat aqueous solution has been reported \cite{kumaran_enigmatic_2000}). A qualitative account has been recently depicted by Luisi \cite{luisi_emergence_2006}, and illustrated by mean of an artificial procedure that intriguingly led to one folded peptide (44 amino acid long) \cite{chessari_production_2006}.

The scenario is based on combinatorial peptide ligation combined with a selection rule that favours only a small number of produced peptides ~~for being~~to b~~ further p~~e processed further~~,~~ along in the next step. In detail, starting from a pool of spontaneously formed peptides, the members of the pool react with each other in combinatorial way by a fragment condensation mechanism. Such reactions can be catalysed by a handful of catalysts, such as Ser-His or other similar compounds, which might be themselves components of the pool or generated after fragment condensation (examples are known of peptide catalysts produced by combinatorial chemistry \cite{berkessel_discovery_2003,wei_enzyme-like_2004}). For example, the full combinatorial coupling of ten 10-mers theoretically gives hundred 20-mers. Next, the produced 20-mers reacts with 10-mers or with 20-mers to give 30-mers or 40-mers, etc. (Figure \ref{fig:fragment_condensation}).

```
\begin{figure}[H]
\centering
\includegraphics[width=12cm]{fragment-condensation}
\caption{Peptide fragment condensation (adapted from  \cite{luisi_emergence_2006}).
Condensation scheme of $n$ peptides (10 amino acids long) to yield ideally $n^2$  peptides (20
amino acids long), of which only $m$ are soluble, and undergo further random fragment
condensation yielding $m^2$ peptides (40
amino acids long), of which only a few will be water soluble. By continuing this procedure, we
reach long chains which are soluble and that can be seen as the product of  a prebiotic molecular
evolution. The asterisk
indicates a catalytic center, capable of inducing peptide synthesis. The synthesis can be in principle
also induced by an external catalytic peptide. Reproduced from \cite{adamala_open_2014} with the
permission of the authors.}
\label{fig:fragment_condensation}
\end{figure}
```

Overlaid to this growth, which quickly increases the peptide length and thus the propensity to fold in a three dimensional compact structures, one can imagine selection rules that reduce the population of peptides participating ~~to~~in the growth, for example by precipitation, degradation, incapacity of binding to the catalysts, or by having partially hidden terminal groups not allowing for

further elongation. Moreover, combinatorial coupling could not involve, with a similar efficiency, all possible reaction partners. For example, peptides that strongly interact with each other, due to multi-point molecular recognition, might be favored over the others. The net result is that given a set of environmental conditions (pH, temperature, salinity, presence of denaturing agents, presence of surfaces), the huge number of potential products is reduced and plausibly converges to few compounds. These will be long, folded peptides with potential catalytic activity, not only toward fragment condensation but also with respect to other reactions.

This hypothetical scenario has not been proved experimentally, but operating by the Merrifield synthesis it has been shown how it could work in practice. Chessari \textit{et al}. \cite{chessari_production_2006} showed that eight random 10-mer peptides, whose random sequences were generated by computers, were divided in two groups and combined to give sixteen 20-mers, which were all synthesized individually and subjected to solubility test. The four more soluble ones were next combined with other four 20-mers to give sixteen 40-mers. Two of these 40-mers, which were partially soluble, were further elongated to the \textit{N}-terminus with a four hydrophilic amino acids tail (DDEE), to give two 44-mers, one of which was soluble in water (Figure \ref{fig:salvo}a). Circular dichroism indicates 22 residues in alpha-helix conformation, in agreement with simple predictions (based on primary sequence analysis. Three-dimensional modeling (ROKKY protein folding suite \cite{ginalski_3d-jury:_2003}) suggests that the peptide folds as three α helices around a hydrophobic core (Figure \ref{fig:salvo}b).

```
\begin{figure}[H]
\centering
\includegraphics[width=13cm]{salvo}
\caption{Novel folded peptide by fragment condensation \cite{chessari_production_2006}.
(\textbf{a}) The sequence of a 44-mer peptide deriving from fragment condensation of 4 random
10-mers, whose sequence was randomly generated. Starting from the 10-mers, a library of 20-mers
was constructed by Merrifield synthesis, and the soluble products were selected for the next
condensation cycle. To the final 40-mer, which was partially soluble, a hydrophilic tail (DDEE) was
added to the \textit{N}-terminus, yielding a soluble 44-mer peptide which was subjected to circular
dichroism investigation. Spectroscopic data indicated that about 50\% of the residues were in
$\alpha$-helix conformation. (\textbf{b}) Model of the three-dimensional structure of the 44-mer
peptide (obtained by the ROKKY protein structure suite). Left: global view; right: detailed view of
the hydrophobic core. Reproduced from Chessari \textit{et al}. \cite{chessari_production_2006}
with the permission of Springer.}
\label{fig:salvo}
\end{figure}
```

The fragment condensation scenario is based on reverse proteolysis (peptide ligation). We have seen that Ser-His, a dipeptide that can be used as a model for primitive protease/peptide ligase

compound, requires the activation of the \textit{C}-terminus. Thus, as we have remarked above, activated molecular partners are necessary for a realistic fragment condensation scenario. Upntil now efficient fragment condensation with the help of Ser-His was only obtained from protected substrates (Figure 5), thus precluding further condensation of fragments. Obviously, for condensation steps to proceed further we need to ensure that reaction schemes described in Figure 5 as "1+1+..." which result in unprotected products advance in more efficient manner. The other related Ser-His requirement is the low solubility of the products, which is needed to shift the equilibrium toward the peptide. This contrasts with the fragment condensation scenario, as the precipitation of a large part of the peptide library is intended as a step to eliminate, rather than selecting, coupling products. However, as it has been demonstrated by Adamala and Szostak \cite{adamala_competition_2013} that if a reaction is carried out in a two-phase system, for example in the presence of lipid vesicles, the lipid bilayers iss are the hydrophobic environment where water-insoluble product can migrate. This possibility actually enriches the fragment condensation scenario by including lipid vesicles (for instance, fatty acid vesicles that are considered the most plausible protocellular structures) and membrane-associated peptides: a fascinating peptide/lipid cooperation.

In this context it should be recalled that interesting studies have shown that molecular crowding, which has been found in some vesicle systems \cite{luisi_spontaneous_2010,pereira_de_souza_spontaneous_2011,stano_remarkable_2013}, favours reverse proteolysis by volume exclusion effects \cite{somalinga_volume_2002}. The lipid membrane can favor growing reactions only inside the liposome lumen, owing to its semi-permeability, and provide at the same time a matrix for the peptide formation \cite{grochmal_modulation_2015}. For example, it has been suggested that hydrophobic amino acids peptides could concentrate at the boundaryon boundaries between lipid domains \cite{raine_lipid_2007}.

The experimental verification of these potentially prebiotic mechanisms is challenging, but complementary information could be achieved by in silico approaches, e.g., molecular modeling combined with stochastic simulations can be helpful to explore populations of random peptides, their condensation, and the outcome of selection rules.

\section{Other potential functions of small peptides}

\label{sect:other}

In modern proteins, cationic peptides, rich in arginine and lysine, are known to strongly interact with RNA and DNA \cite{tan_structural_1995,derouchey_comparison_2013}. Nuclear DNA binding histones and protamines are mostly composed of such cationic proteins \cite{balhorn_dna_2000,bartova_histone_2008}.

Shorter oligopeptides rich in Arg and Lys residues are also capable of interactions with nucleic acids \cite{tan_structural_1995,mascotti_thermodynamics_1997}. For example, Arg-Arg-Arg tripeptides can aid DNA condensation via electrostatic interactions \cite{derouchey_comparison_2013}. Poly-arginine and poly-lysine residues form salt bridges with nucleic acid and provide charge shielding of the nucleic acid backbones \cite{ghosh_role_2003}.

Arginine-rich peptides are prebiotically plausible, with several routes of the synthesis of arginine under prebiotic conditions \cite{liu_polymerization_1998,xin_6-membered_2009}. It is possible that the cationic peptides could have stabilized fold, and therefore aid function, of the nucleic acids employed in the early life metabolism.

The emergence of self-replicating nucleic acids likely required some form of templating ~~of~~ of nucleic acid synthesis \cite{szostak_eightfold_2012}. One obvious templating surface in a protocell would be a bilayer membrane. However, both RNA backbone and lipid headgroups are negatively charged –- making direct templating interactions impossible. It has been recently shown that hydrophobic peptides can promote localization of RNA to the liposome membrane, therefore providing templating surfaces for RNA activity \cite{kamat_electrostatic_2015}.
Small hydrophobic peptides themselves can also interact with the lipid bilayer membranes. A small hydrophobic peptide AcPheLeuNH$_2$ can localize into the fatty acid bilayer membranes. This causes the vesicles containing the peptide to grow~~th~~ at the expense of vesicles without the peptide. Furthermore, AcPheLeuNH$_2$ can be synthesized by the Ser-His dipeptide. Combined, this creates a system where encapsulated small peptide catalysts can impact the fitness of model protocells, providing insight into the emergence of~~ mechanism of~~ Darwinian selection \cite{adamala_competition_2013}.

Amino acids, especially neutral and hydrophobic ones, can cross simple, prebiotically plausible fatty acid membranes \cite{chakrabarti_permeability_1992}. However, as early life moved from leaky and unstable fatty acid membranes to the modern phospholipid membranes, the permeability decreased sharply \cite{deamer_chemistry_2005}. In the absence of active membrane transport, this created a problem of delivery of substratesin substrate delivery for the synthesis of peptides inside the protocell. It has been demonstrated that pH-gradients mediated amino acid transport providesand provided a solution to this problem \cite{chakrabarti_permeability_1992}. This suggests that protocell fatty acid membranes and encapsulated catalysts could have co-evolved since the earliest stages of the history of life \cite{a_wilson_towards_2014}.

By using a model system (Boc-tryptophan pyranine ester as acyl donor and tryptophan benzylamide as nucleophile), it was shown that the selective permeability of liposome membrane, intrinsically facilitateing the accumulation of condensation products inside liposomes \cite{grochmal_modulation_2015}-. Moreover, it has been suggested that boundaryies between lipid domains might lead to peptide accumulation, leading to optimal conditions for further peptide reactions \cite{raine_lipid_2007}.

\section{Looking for short random primordial peptides with catalytic functions}
\label{sect:looking_for}
The role of amino acids as catalysts for some chemical reactions has been greatly emphasized in synthetic organic chemistry with the advent of organocatalysis, and recent studies point to short catalytic peptides as minimalistic form of enzymes \cite{duncan_short_2015}, revealing the potential of the still unexplored huge pool of short random peptides in organic synthesis and in prebiotic chemistry scenarios.

Ser-His, whose activity has been discussed in detail in this review, is just an example of these to-be-discovered peptides. It should be remarked that histidine is not generally considered a prebiotic amino acid. However, its synthesis in simulated prebiotic conditions has been also reported \cite{shen_prebiotic_1990}. Although the detailed mechanism of Ser-His activity has not been clarified yet, this does not weaken the range and the relevance of the reported reactions. In particular, the fact that a simple dipeptide can catalyse, even if with low efficiency, an important class of reactions for prebiotic chemistry is a strong message suggesting further investigations in the field of peptide organocatalysis.

Peptides whose length is between 10 and 40 residues (and more) can be formed –- at least in principle –- by spontaneous polymerisation of activated amino acids followed by fragment condensation, with the help of short peptide catalysts that are themselves components of the peptide pool. Not much is known about co-oligomerization of amino acids to form short peptides (e.g., up to the 10-mer) and about the nature of the resulting sequences (for example, see \cite{lamy_glutamateglycine_2008,schwab_papain_2012}). Are these really random? ~~How~~ What did early peptides look~~ed~~ like? Peptides, in contrary to nucleic acids, are stable and years-persistent molecules in the absence of catalysts (the half-life for ~~the~~ uncatalyzed peptide hydrolysis has been estimated to be of 450 years \cite{wolfenden_depth_2001,miller_catalytic_2002}. The gap between Miller-type experiments, referring to the accumulation of monomeric building blocks and even moderately complex scenarios with systems of molecules interacting in diverse ways, ~~maybe~~ perhaps in the presence of membranes, is actually huge. In this context, we are still missing not only ~~evidences from experiments~~experimental evidence, but also a conceptual framework.

On the other hand, the exploration of fully random peptide libraries, looking for folding and catalysis, is challenging due to the vastness of the sequence space and to the peculiar physico-

chemical behaviour of peptides, which is hardly predictable. Attempts to ~~enquire a~~ acquire a library of 10^9 random 50-mer peptides have been reported, resulting in a sub-population of peptides which are partially resistant to thrombin digestion, hence potentially folded \cite{chiarabelli_investigation_2006-I,chiarabelli_investigation_2006-II}. Other reports also suggest that peptides formed by random sequences \cite{doi_high_2005}, or polar/non-polar alternate sequences \cite{kamtekar_protein_1993}, or from a reduced amino acid~~s~~ alphabet \cite{davidson_cooperatively_1995} can fold in well defined three dimensional structures, hence being potentially catalytic. Also, ~~it should not be discarded~~ the possibility of peptides which have function only when assembled to each other \cite{rufo_short_2014,singh_towards_2015} should not be discarded.

The results and the considerations presented here, therefore, actually call for further theoretical, experimental, and in silico investigations on short peptides with random amino acid sequences –- an enormous reservoir of molecular diversity. Finding new catalytic peptides would be of significance for understanding the origin of life and it can also have a potentially high impact in synthetic chemistry and biotechnology.

\textbf{Addendum.} While writing this review we have discovered a very recent paper where the esterase/peptidase Ser-His activity has been re-examined and questioned based on additional tests \cite{macdonald_evaluation_2016}. In addition \textit{p}-nitrophenylacetate, the authors have assayed the Ser-His hydrolytic function with fluorescein esters and rhodamine amides at pH 6, reporting a non-statistically significant difference between control and Ser-His containing samples. \\
